# Innate Immunomodulatory Activity of Cedrol, a Component of Essential Oils Isolated from *Juniperus* Species

**DOI:** 10.3390/molecules26247644

**Published:** 2021-12-16

**Authors:** Gulmira Özek, Igor A. Schepetkin, Moldir Yermagambetova, Temel Özek, Liliya N. Kirpotina, Shyryn S. Almerekova, Saule I. Abugalieva, Andrei I. Khlebnikov, Mark T. Quinn

**Affiliations:** 1 Department of Pharmacognosy, Faculty of Pharmacy, Anadolu University, Eskisehir 26470, Turkey; gulmiraozek@gmail.com (G.Ö.); temelozek@gmail.com (T.Ö.); 2 Department of Microbiology and Cell Biology, Montana State University, Bozeman, MT 59717, USA; igor@montana.edu (I.A.S.); liliya@montana.edu (L.N.K.); 3 Institute of Plant Biology and Biotechnology, Almaty 050040, Kazakhstan; ermaganbetova.moldir@bk.ru (M.Y.); almerekovakz@gmail.com (S.S.A.); absaule@yahoo.com (S.I.A.); 4 Faculty of Biology and Biotechnology, Al-Farabi Kazakh National University, Almaty 050040, Kazakhstan; 5 Medicinal Plant, Drug and Scientific Research and Application Center (AUBIBAM), Anadolu University, Eskişehir 26470, Turkey; 6 Kizhner Research Center, Tomsk Polytechnic University, 634050 Tomsk, Russia; aikhl@chem.org.ru

**Keywords:** *Juniperus*, essential oil, calcium flux, neutrophil, chemotaxis, sesquiterpene, cedrol

## Abstract

Little is known about the immunomodulatory activity of essential oils isolated from *Juniperus* species. Thus, we isolated essential oils from the cones and leaves of eight juniper species found in Montana and in Kazakhstan, including *J. horizontalis*, *J. scopolorum*, *J. communis*, *J. seravschanica*, *J. sabina*, *J. pseudosabina*, *J. pseudosabina* subsp. *turkestanica*, and *J. sibirica*. We report here the chemical composition and innate immunomodulatory activity of these essential oils. Compositional analysis of the 16 samples of Juniper essential oils revealed similarities and differences between our analyses and those previously reported for essential oils from this species. Our studies represent the first analysis of essential oils isolated from the cones of four of these Juniper species. Several essential oil samples contained high levels of cedrol, which was fairly unique to three Juniper species from Kazakhstan. We found that these essential oils and pure (+)-cedrol induced intracellular Ca^2+^ mobilization in human neutrophils. Furthermore, pretreatment of human neutrophils and *N*-formyl peptide receptor 1 and 2 (FPR1 and FPR2) transfected HL60 cells with these essential oils or (+)-cedrol inhibited agonist-induced Ca^2+^ mobilization, suggesting these responses were desensitized by this pretreatment. In support of this conclusion, pretreatment with essential oils from *J. seravschanica* cones (containing 16.8% cedrol) or pure (+)-cedrol inhibited human neutrophil chemotaxis to *N*-formyl peptide. Finally, reverse pharmacophore mapping predicted several potential kinase targets for cedrol. Thus, our studies have identified cedrol as a novel neutrophil agonist that can desensitize cells to subsequent stimulation by *N*-formyl peptide.

## 1. Introduction

The genus *Juniperus* L. consists of 75 species, which grow mainly in the northern hemisphere [1]. *Juniperus* is one of the main genera of the Cupressaceae family [2], and according to the generally accepted classification, the genus is divided into three sections distributed throughout the world [1,3,4]. These evergreen coniferous trees or shrubs thrive under a variety of environmental conditions. Some juniper species grow in the Arctic zone and the alpine belt, while others are found in subtropical and tropical zones [1,5,6,7,8].

Among the least studied *Juniperus* are those growing in Kazakhstan. In Kazakhstan, the local names for *Juniperus* species are “arsha” (in Kazakh) and “mojjevelnik” (in Russian) [9,10]. Kazakhstan’s latitude ranges from 40°7′ N to 54°9′ N, and therefore, is geographically similar to the northern states of the USA, including Montana (44°2′ N to 49°0′ N). As in Kazakhstan, a large part of Montana is occupied by steppes (approx. 60%), deserts, and semi-deserts (20%). About 10% of both Montana and Kazakhstan are covered by mountain systems where *Juniperus* species are an important woodland component [11,12,13]. According to the Flora of Kazakhstan [14], there are 10 *Juniperus* species in this country (*J. communis* L., *J. sibirica* Burgsd., *J. pseudosabina* Fisch. et. C. A. Mey., *J. turkestanica* Kom., *J. intermedia* Drob., *J. seravschanica* Kom., *J. sabina* L., *J. semiglobosa* Regel, *J. talassica* Lipsky and *J. drobovii* Sumn.). However, further studies by Abdulina [15] reduced them to seven species (*J. communis, J. × media, J. pseudosabina, J. sabina, J. semiglobosa, J. sibirica* and *J. seravschanica*), which was supported by molecular taxonomy indicating that *J. turkestanica* and *J. pseudosabina* belonged to a single species named *J. pseudosabina* [12]. Therefore, the former species was renamed as *J. pseudosabina* subsp. *turkestanica*. In Kazakhstan, juniper is one of the important components of forests in the high mountain ranges of Altai, Tarbagatai, Dzhungarskiy Alatau, Tien Shan, and in the boron forests of Northern Kazakhstan [16,17,18,19]. In comparison, there are three *Juniperus* species native to the state of Montana, USA (*J. communis*, *J. horizontalis*, *J. scopulorum*) [1]. Hence, only *J. communis* is common to both Kazakhstan and Montana. In both regions, the soil composition depends on climatic conditions and changes with variations in geography. Accordingly, the chemical composition of the vegetation also changes. Therefore, a comparative assessment of the chemical composition of juniper species growing in various eco-geographical niches of Kazakhstan and Montana is important to understanding their potential as sources of novel therapeutics, such as essential oils.

*Juniperus* species are a source of essential oils that have been used in the cosmetic industry. Essential oils serve as a source of aromatic substances to improve perfume properties and increase resistance to oxidation [20,21]. Juniper oil is also used in medicine as an effective antiseptic [22,23]. Besides essential oils, juniper extracts are rich in lipids, alkaloids, glucosides, polyphenols, and ascorbic acid. Juniper needles contain a significant amount of chlorophylls and carotenoids, which have antioxidant properties [24,25,26]. Representatives of the genus *Juniperus* have also been used in traditional medicine, gastronomy [27], pharmaceuticals [28], and in alcoholic beverages [29,30].

In traditional medicine, extracts from *Juniperus* species have been used for their stomachic, diuretic, antiseptic, antirheumatic, antispasmodic, astringent, carminative, and rubificant properties to treat dyspepsia, cystitis, arthritis, gout, and other inflammatory conditions [31,32,33]. One of the most important representatives of the genus is *J. communis* L., and the dried ripe cones of *J. communis* and essential oils obtained by steam distillation from the ripe, non-fermented cones of *J. communis* are included in the European Pharmacopoeia [34]. In addition a monograph about Juniper Tar, which is the empyreumatic volatile oil obtained from the woody portions of *J. oxycedrus* L., is registered in the United States Pharmacopoeia [35]. Juniper cones are the source of essential oils and the subjects of Pharmacopoeial monographs in the 5th Polish National Pharmacopoeia [36], German Pharmacopoeia (Deutches Arzneibuch) [37] and in Martindale Complete Drug Reference [38].

The essential oil composition of *Juniperus* species around the world have comprehensively been evaluated by Adams et al. [1,39,40,41,42,43,44,45]. However, reports on essential oils from *Juniperus* species growing in Kazakhstan have focused mainly on the investigation of essential oil yields and major volatile compounds isolated from their leaves [10,39,41,43,45,46,47,48], and little information has been reported regarding their biological properties [9,10,49,50].

The chemical composition of essential oils isolated from different *Juniperus* species is characterized by large variation, and it is difficult to make any common classification or grouping. In general, these essential oils are characterized by a high abundance of monoterpene hydrocarbons, mostly α-pinene, sabinene, myrcene, and limonene [41,43,44,45,47,51,52,53,54,55,56,57,58,59,60,61,62,63,64,65,66,67,68,69,70,71,72,73,74,75,76,77,78]. In addition, oxygenated monoterpenes, such as linalool, bornyl acetate, and sabinyl acetate, were also found in the essential oils of several *Juniperus* species [10,46,62,64]. Sesquiterpene constituents are rare in *Juniperus* oils [71], whereas diterpenes (manoyl oxide, abietatriene) were reported only in Algerian juniper essential oils [67]. Thus, it is clear that a broader evaluation of the chemical composition and biological activity of essential oils from plants found in various regions of the world is necessary to expand our understanding of the regional differences in these species.

In the present studies, we compared the chemical composition and immunomodulatory activity of essential oils obtained from the cones and leaves of seven juniper species collected in Kazakhstan and Montana, including *J. horizontalis*, *J. scopolorum*, *J. communis*, *J. seravschanica*, *J. sabina*, *J. pseudosabina*, *J. pseudosabina* subsp. *turkestanica*, and *J. sibirica* and analyzed their chemical composition and innate immunomodulatory activity. We show that the Juniper essential oils inhibited intracellular Ca^2+^ mobilization in neutrophils. Furthermore, we demonstrated that cedrol, which was present at high levels in some Juniper species from Kazakhstan, also inhibited human neutrophil functional responses and is likely one of the active components in these essential oils. Given the critical role of neutrophils in inflammation, our data support the possibility that cedrol could be effective therapeutic compound for the development anti-inflammatory agents.

## 2. Results and Discussion

### 2.1. Essential Oil Composition

Leaves and cones were collected from mature *Juniperus* shrubs and trees (Table 1) and extracted, as described under Materials and Methods. The extraction yields (*v/w*) of essential oils obtained from *Juniperus* spp. ranged from 0.4 to 2.0% (Table 1). The chemical composition of these essential oils was evaluated using simultaneous GC-FID and GC/MS. Major compounds (>2%) of the essential oils are shown in Table 2, and Supplementary Table S1 summarizes all identified compounds. The main class of compounds in all samples was monoterpenes, which ranged from 52.5 to 98.3% of the essential oil composition isolated from various Juniper species (Table 3). In addition, essential oils from the cones of *J. sibirica* (JSI_Con_) were enriched in sesquiterpenes (46.4%).

The leaf oils of *J. horizontalis* from Turkey, Iran, USA, Egypt, and Canada (Saskatchewan) have been previously reported to contain linalool, limonene, sabinene, and bornyl acetate as their major volatile constituents [44,62,63,64,79]. In the present study, we found that JH_Lv_ also had a high content of sabinyl acetate (>16%), which was not found in previous reports (Table 2). The cone oil of *J. horizontalis* from Iran was reported to contain myrcene (22.6%), limonene (27.8%), and sabinene (38.0%) [79]. In our investigation, JH_Con_ had predominantly sabinene (>77.0%) (Table 2), which may reflect the differences in ecology where these plants were collected.

We found that *J. scopulorum* essential oils JS_Lv_ and JS_Con_ had high contents of sabinene (37.6% and 35.8%, respectively) (Table 2). These data are consistent with previous reports [80,81]. It should be noted that JS_Lv_ and JS_Con_ essential oils also had high levels of terpinen-4-ol (10.0% and 23.0%, respectively). We also found that the eudesmane group of sesquiterpenes was enriched in the leaf oils of *J. scopulorum*, including elemol (14.4% and 6.0%, respectively) and 8-α-acetoxyelemol (11.0% and 1.0%, respectively) (Table 2). The co-occurrence of pregeijerene B in *Juniperus* oils was shown to be highly correlated with 8-α-acetoxyelemol and has systematic significance for this genus [82]. Indeed, we also detected pregeijerene B (2.2%) in the leaf oil of *J. scropulorum.* Similarly, JS_Con_ also had similar amounts of 8-α-acetoxyelemol (1.0%) and pregeijerene B (0.2%). Previously, leaf oils of *J. scopulorum* from Colorado (USA) were reported to contain 8-α-acetoxyelemol (8.7%) and elemol (3.1%) [83], and *J. scopulorum* leaf oil from New Mexico was reported to contain thujopsene (57.9%), cuparene (6.1%), and cedrol (6.1%) [79]. Likewise, we found that JS_Lv_ essential oils had high levels of sesquiterpenes (Table 2 and Table 3).

*J. communis* is one of the most studied *Juniperus* species, and our data confirmed the relatively high content of α-pinene in the leaf oil of *J. communis* [51,52,54,55]. In addition, *J. communis* cone oil contained a high amount of myrcene (53.2%), which is consistent with previous reports on essential oils from *J. communis* collected in Poland [52] (Table 2).

The essential oils of *J. seravschanica* (JSE_Lv_ and JSE_Con_) were characterized by a high content of α-pinene (45.3% and 34.4%, respectively), myrcene (19.7% and 19.5%, respectively), and limonene (3.2% and 4.5%, respectively) (Table 2). The sesquiterpene alcohol cedrol (13.1% and 16.8%, respectively) was also present in significant amounts. These data are consistent with previously published information about *J. seravschanica* leaf oils from Kazakhstan, Pakistan, and Iran [42,45]. Similarly, we found that the cone essential oil JSE_Con_ also contained monoterpenes, α-pinene (34.4%), myrcene (19.5%), and limonene (4.5%), as well as cedrol (16.8%) (Table 2). In addition, the diterpene 8,13-abietadiene (3.9%) was also present in JSE_Con._ Consistent with our findings, *J. seravschanica* cone oil from Uzbekistan was previously reported to contain α-pinene (29.0%), germacrene B (5.9%), and cedrol (3.1%) [73].

Analysis of *J. sabina* leaf essential oil composition showed that they were enriched in sabinyl acetate (30.3%), cedrol (12.2%), and sabinene (33.2%), which is consistent with previous reports [10,46]. Analysis of the *J. sabina* cone essential oils, which has not been performed previously, showed that they were dominated by sabinene (74.1%) (Table 2).

Sabinene (39.6%), α-pinene (15.2%), terpinen-4-ol (9.5%), and linalool (3.6%) were found to be the main constituents in *J. pseudosabina* subsp. *turkestanica* leaf essential oils (JT_Lv_), which confirmed previous reports [43,47] (Table 2). *J. pseudosabina* subsp. *turkestanica* cone essential oils (JT_Con_), which have not been previously reported, were enriched in sabinene (54.2%), α-pinene (25.1%), and myrcene (4.8%) (Table 2).

The leaf essential oils of *J. pseudosabina* from Kazakhstan, China, and Mongolia have been reported to contain α-pinene, sabinene and cedrol, as well as high levels of germacrene-D-4-ol (14.3%) in plants from Mongolia [43,56]. Consistent with these reports, we found that JPS_Lv_ were enriched in monoterpenes, α-pinene (30.8%), sabinene (19.4%), and terpinen-4-ol (4.0%), as well as cedrol (7.0%) and elemol (3.9%) (Table 2). The cone essential oils of *J. pseudosabina* (JPS_Con_) have not been analyzed previously and were enriched in α-pinene (49.3%), sabinene (20.4%), myrcene (4.1%), β-pinene (3.7%), and cedrol (3.4%) (Table 2).

Leaf essential oils of *J. sibirica* from Italy, Kazakhstan, Serbia, and Russia have been reported to contain high levels of α-pinene (up to 80.0%) [46,74,75,76]. In addition, Sampietro et al. [48] reported that leaf essential oils leaf essential oils of *J. sibirica* from Kazakhstan were enriched in α-thujene-rich (46.3%). Here, we found that JSI_Lv_ contained α-pinene (26.9%), sabinene (24.3%), terpinen-4-ol (9.3%), myrcene (2.6%), and α-terpinyl acetate (2.7%), whereas JSI_Con_ contained mainly high levels of α-pinene (44.9), as well as significant levels of germacrene D (16.7%) and 1,6-germacradien-5β-ol (4.2%) (Table 2). In addition, δ-cadinene (5.8%) was present. The only other report on *J. sibirica* cone essential oils was based on plants from Russia and indicated the presence of α-pinene (19.9%), β-myrcene (5.0%), α-terpineol (6.0%), as well as α-bisabolene (6.1%), τ-cadinol (5.6%), and α-cadinol (6.7%) [75]. Thus, there are clear similarities and differences with our analyses, which again may be related to the geographical location or time of collection of the plant tissues analyzed.

### 2.2. Effect of the Juniperus Essential Oils and Cedrol on Neutrophil [Ca^2+^]_i_

The innate immune system is essential for host defense and provides immediate defense against infection. Neutrophils are the most abundant leukocytes in the blood and constitute key components of the innate immunity that perform an important effector and regulatory functions in the development of human inflammatory response [84,85]. Thus, neutrophils represent an ideal pharmacological target for therapeutic development, and numerous natural products, including essential oils, have been shown to exhibit neutrophil immunomodulatory activity [86,87,88].

**Figure 1:**
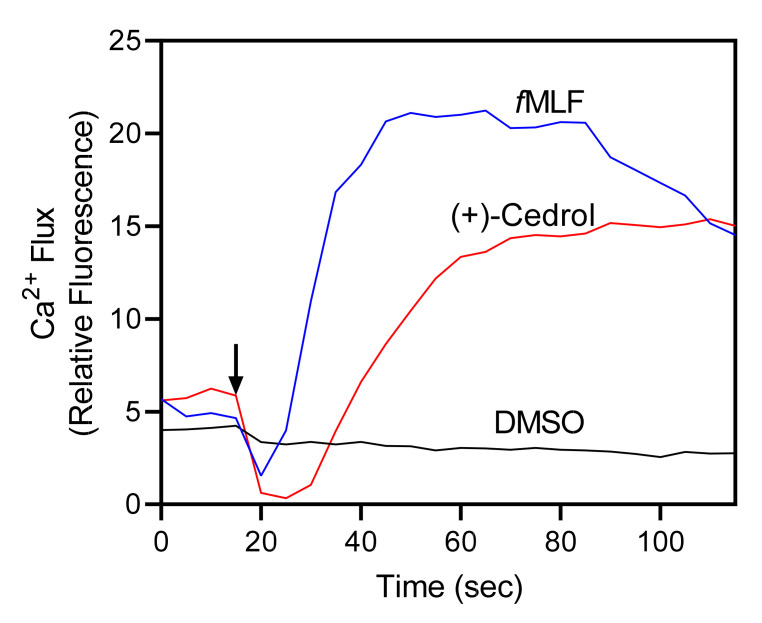
(+)-Cedrol induces neutrophil Ca^2+^ mobilization. Human neutrophils were treated with 25 µM (+)-cedrol, 5 nM *f*MLF (positive control), or 1% DMSO (negative control), and [Ca^2+^]_i_ was monitored for the indicated times (arrow indicates when treatments were added). The data are from one experiment that is representative of three independent experiments.

**Figure 2:**
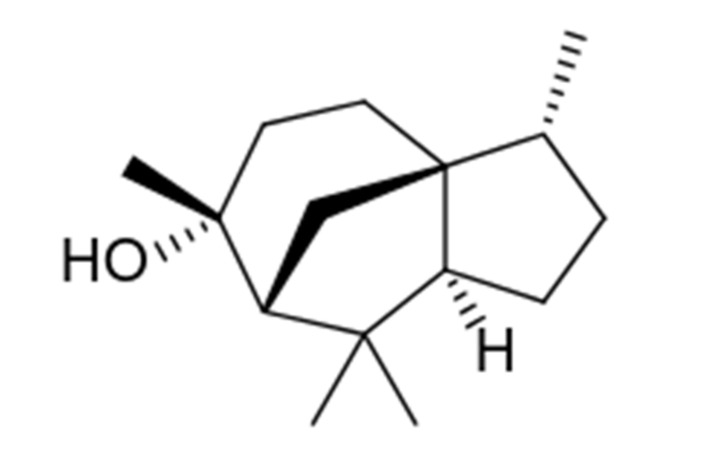
Chemical structure of (+)-cedrol.

*Juniperus* essential oils were evaluated for their immunomodulatory effects on human neutrophils. Specifically, we evaluated their effects on [Ca^2+^]_i_, which is a key component of phagocyte activation [45,46]. We found that treatment of neutrophils with *Juniperus* essential oils increased [Ca^2+^]_i_, with EC_50_ values ranging from 13.9 µg/mL (JT_Con_) to 49.8 µg/mL (JH_Con_). In addition, analysis of (+) cedrol, a sesquiterpene present at levels of 13.1% (JSE_Lv_) to 16.8% (JSE_Con_) showed that this compound also activated neutrophil [Ca^2+^]_i_ (Figure 1 and Table 4). Thus, cedrol represents a novel essential oil component with innate immunomodulatory activity. Note that we evaluated (+)-cedrol (Figure 2), because this isoform is present in *Juniperus* essential oils [89].

**Figure 3:**
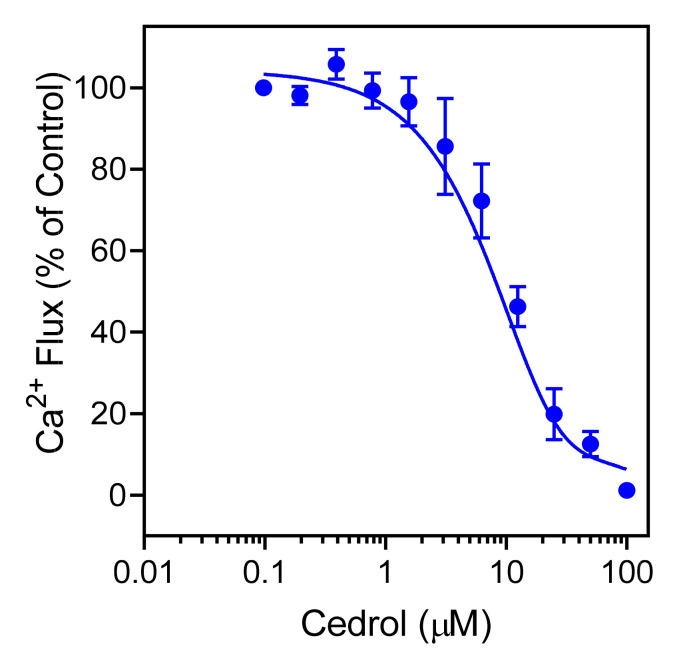
Inhibition of *f*MLF-induced neutrophil Ca^2+^ mobilization by (+)-cedrol. Human neutrophils were treated with the indicated concentrations of (+)-cedrol or 1% DMSO (negative control) for 10 min. The cells were activated by 5 nM *f*MLF, and [Ca^2+^]_i_ was monitored, as described. The data are from one experiment that is representative of three independent experiments.

Since *Juniperus* essential oils and cedrol stimulated human neutrophil [Ca^2+^]_i_, and it is well recognized that agonists can down regulate neutrophil responses to subsequent treatment with heterologous or homologous agonists [90], we evaluated whether *Juniperus* essential oils and/or cedrol could inhibit agonist-induced Ca^2+^ mobilization in human neutrophils and FPR-transfected HL60 cells. As shown in Table 4, *Juniperus* essential oils inhibited [Ca^2+^]_i_ in *f*MLF-stimulated FPR1-HL60 cells and in WKYMVM-stimulated FPR2-HL60 cells with IC_50_ values in the micromolar range. Most of the essential oils also inhibited *f*MLF-induced Ca^2+^ flux in human neutrophils. However, essential oils from *J. horizontalis* cones (JH_Con_) were inactive. Note that JH_Con_ has a minimal content of sesqueterpenes (Table 2 and Table 3), which seem to contribute to the observed biological activities of the other *Juniperus* essential oils. Analysis of cedrol, the unique sesquiterpene in essential oils from *J. seravschanica*, *J. sabina*, and *J. pseudosabina* (Table 2) showed that it also inhibited *f*MLF-induced neutrophil [Ca^2+^]_i_ (Table 4), and this effect was dose-dependent (Figure 3), indicating that cedrol desensitized the neutrophils and FPR-transfected HL60 cells to subsequent agonist activation.

Previously, we analyzed the effects of a number of essential oil compounds on human neutrophil Ca^2+^ flux and found that many of the same compounds that are present in *Juniperus* essential oils, including α-pinene, β-pinene, sabinene, myrcene, α-terpinene, limonene, γ-terpinene, *p*-cymene, linalol, and terpinene-4-ol, had no effect on human neutrophils [86,87]. The only exception was germacrene D, which is present in JC_Con_, JC_Lv_, and JSI_Con_ and was shown previously to active human neutrophil [Ca^2+^]_i_ [91]. On the other hand, these are the first studies to evaluate the effects of cedrol on neutrophil function.

### 2.3. Effect of Cedrol on Neutrophil Chemotaxis

**Figure 4:**
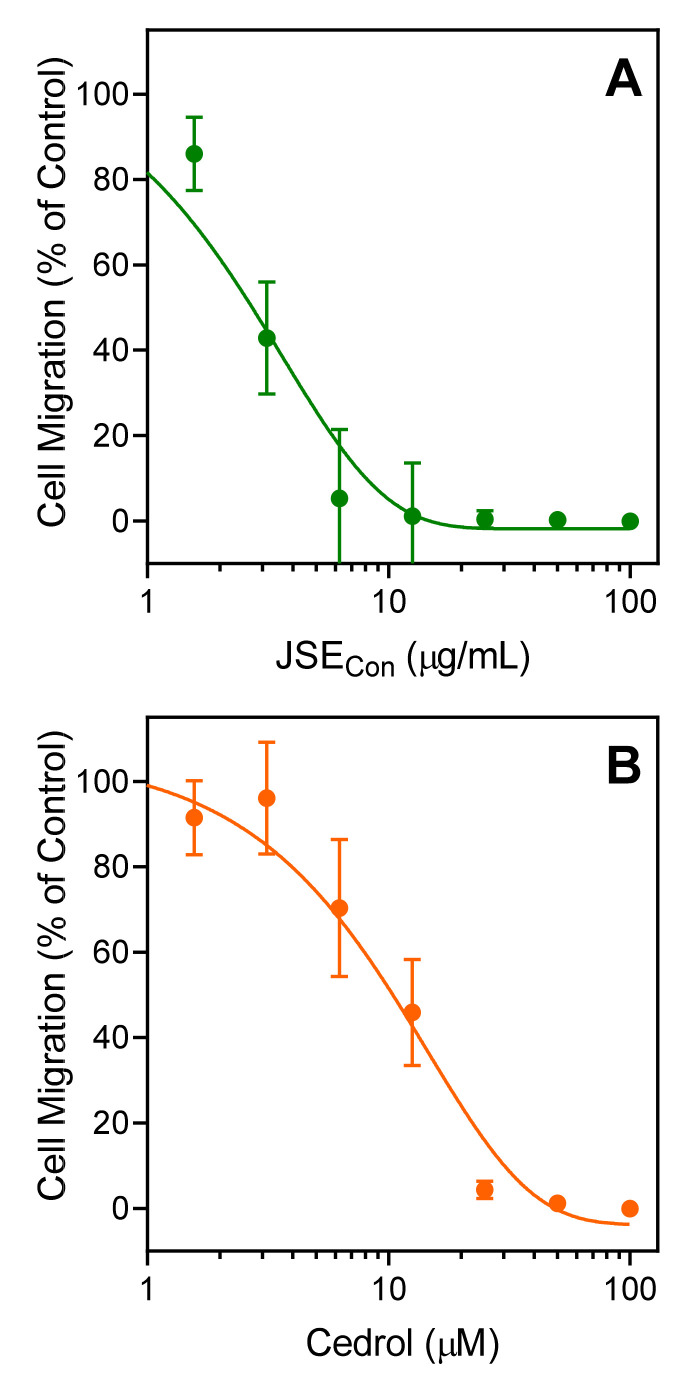
Inhibition of neutrophil chemotaxis by essential oil *J. seravschanica* cones (JSE_Con_) (**A**) and (+)-cedrol (**B**). Neutrophil migration toward 1 nM *f*MLF was measured, as described under *Materials and Methods*. The data are from one experiment that is representative of two independent experiments.

Various essential oils and their components have been reported previously to inhibit neutrophil migration [86,87,91]. We found that pretreatment with JSE_Con_ for 10 min dose-dependently inhibited *f*MLF-induced human neutrophil chemotaxis, with an IC_50_ of 3.1 ± 1.1 µg/mL (Figure 4A). Likewise, cedrol also inhibited *f*MLF-induced neutrophil chemotaxis, with an IC_50_ of 10.6 ± 3.4 µM (Figure 4B). These results are consistent with the ability of these treatments to inhibit agonist-induced [Ca^2+^]_i_ in human neutrophils and again support the conclusion that cedrol is a novel innate immunomodulator.

**Figure 5:**
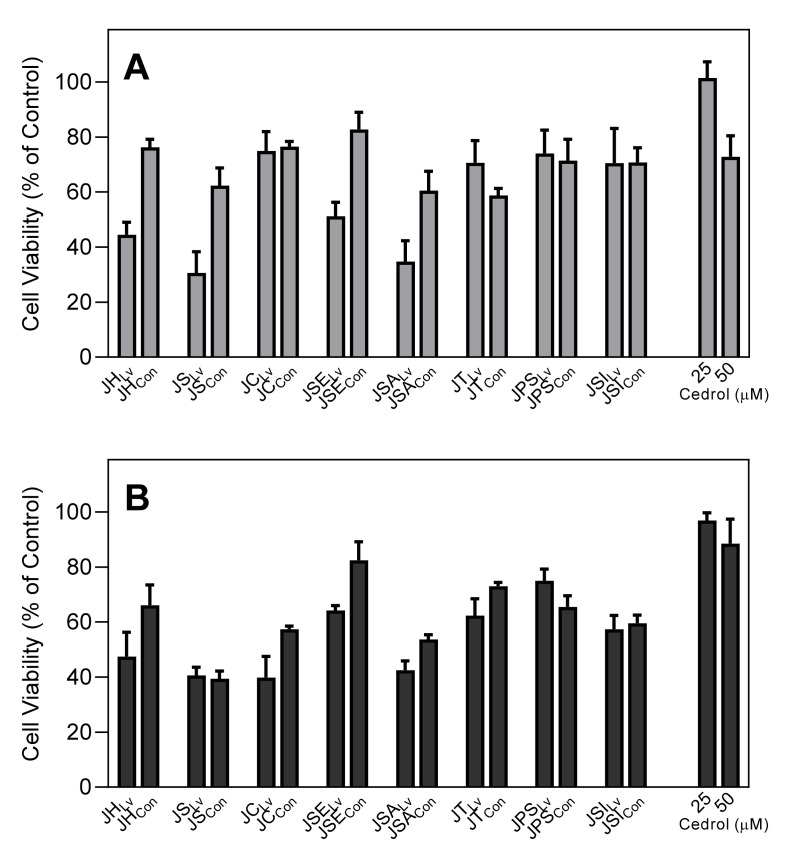
Cytotoxicity of *Juniperus* essential oils and cedrol. HL60 cells were preincubated with 25 µg/mL of Juniper oil or 25 and 50 µM of pure (+)-cedrol for 30 min (**A**) and 90 min (**B**) and cell viability was analyzed, as described. Values are the mean ± SD of triplicate samples from one experiment that is representative of two independent experiments with similar results.

To ensure that our analyses of neutrophil functional activity were not due to toxic effects, we evaluated cytotoxicity of the essential oils at a fairly high concentration (25 µg/mL) and cedrol (25 and 50 µM) in HL60 cells during 30 min and 90 min incubation periods. These incubation periods are comparable to the times used to measure Ca^2+^ mobilization (up to 30 min) and cell migration (up to 90 min). As shown in Figure 5, some of the leaf essential oils had fairly high cytotoxic effects, especially during longer incubation times (e.g., JH_Lv_, JS_Lv_, and JSE_Lv_). However, cedrol and JSE_Con_, the essential oil containing the highest concentration of cedrol, had little cytotoxicity after 30 or 90 min, verifying the absence of their cytotoxicity during the Ca^2+^ flux and chemotaxis assays.

### 2.4. Identification of Potential Protein Targets for Cedrol

Cedrol has been reported to exhibit a number of biological activities, including anticancer [92,93,94] and anti-inflammatory. For example, cedrol was shown to have analgesic and anti-inflammatory effects in complete Freund’s adjuvant (CFA)-induced arthritis in rats [95] and in mice with collagen-induced arthritis (CIA) [96]. This sesquiterpene also attenuated neuropathic pain in chronic constriction injury of rats by inhibiting the inflammatory response [97]. Despite the various biological activities reported for cedrol, little is known about its specific cellular targets. Thus, we performed reverse-pharmacophore mapping on the molecular structures of (+)-cedrol to identify potential biological targets. PharmMapper was used to compare a large database of pharmacophore patterns with these compounds and generate target information, including normalized fitness scores and pharmacophoric characteristics. It is important to submit a compound to the PharmMapper server in the form of the proper optical isomer, as this methodology explicitly accounts for 3D structure of a molecule. Thus, we evaluated the (+)-configuration of cedrol, which is the most common enantiomer found in *Juniper* plants [89]. The results of PharmMapper analysis indicated that three kinases could be potential targets for cedrol: proviral integration Moloney virus kinase (PIM1), vascular endothelial growth factor receptor 2 (VEGFR2), and c-Jun N-terminal kinase 1 (JNK1) (Table 5). They are present among the 20 top-ranked targets found by PharmMapper.

**Figure 6:**
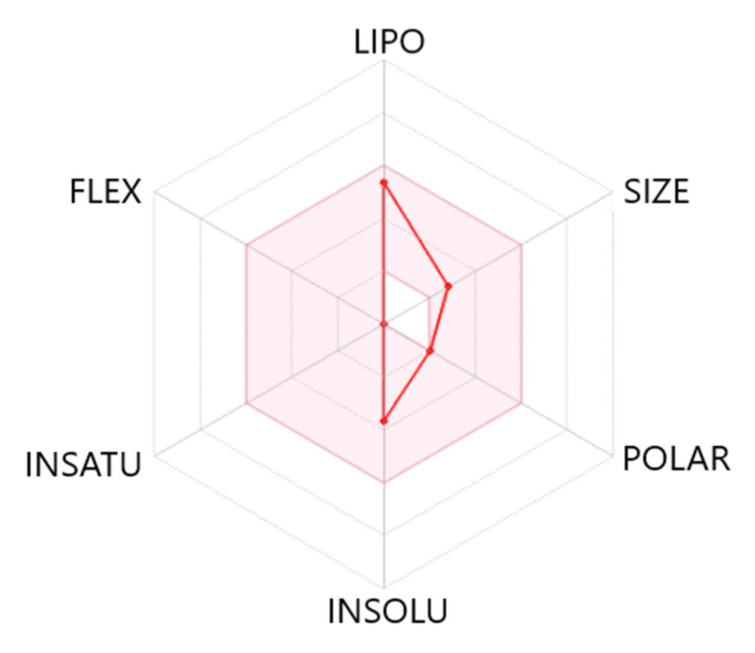
Bioavailability radar plot of (+)-cedrol. The plot depicts the LIPO (lipophilicity), SIZE (molecular weight), POLAR (polarity), INSOLU (insolubility), INSATU (unsaturation), and FLEX (rotatable bond flexibility) parameters.

We also calculated the most important physico-chemical parameters for cedrol using SwissADME [98] (Table 6 and Figure 6) and found that cedrol would be predicted to permeate the blood–brain barrier (BBB). According to the radar plot, the ADME characteristics of (+)-cedrol predict that is would exhibit high bioavailability.

## 3. Materials and Methods

### 3.1. Plant Material

*J. sabina*, *J. pseudosabina*, *J. sibirica*, *J. pseudosabina* subsp. *turkestanica* Kom., *J. communis*, and *J. horizontalis* are evergreen shrubs; *J. seravschanica* and *J. scopolorum* are evergreen trees. Leaves and cones were collected in 2019–2021 from mature shrubs and trees in several gorges of the Trans-Ili Alatau (Almaty region, South-Eastern Kazakhstan), Western Tien-Shan (Turkistan region, Southern Kazakhstan), Tarbagatai mountain range (Eastern Kazakhstan), and Bozeman (MT, USA) (Table 1). The collected samples were air-dried for 7–10 days at room temperature away from direct sunlight before hydrodistillation. Botanical identification of the plant material collected in Kazakhstan was performed by botanist Anna A. Ivashchenko from Ile-Alatau National Park, Almaty, Kazakhstan, who has an extensive experience in the taxonomical identification of *Cupressaceae* species. Botanical identification of the plant material collected in Montana was performed by botanist Robyn A. Klein from Montana State University, Bozeman, MT, USA.

### 3.2. Materials

Dimethyl sulfoxide (DMSO), *N*-formyl-Met-Leu-Phe (*f*MLF), Trp-Lys-Tyr-Val-Met (WKYMVM), and Histopaque 1077 were purchased from Sigma-Aldrich Chemical Co. (St. Louis, MO, USA). (+)-Cedrol was from TargetMol (Boston, MA, USA). *n*-Hexane was purchased from Merck (Darmstadt, Germany). Fluo-4AM was purchased from Invitrogen (Carlsbad, CA, USA). Roswell Park Memorial Institute (RPMI) 1640 medium and Dulbecco’s Modified Eagle’s Medium (DMEM):F12 medium were purchased from HyClone Laboratories (Logan, UT, USA). Fetal calf serum and fetal bovine serum were purchased from ATCC (Manassas, VA, USA). Hanks’ balanced salt solution (HBSS; 0.137 M NaCl, 5.4 mM KCl, 0.25 mM Na_2_HPO_4_, 0.44 mM KH_2_PO_4_, 4.2 mM NaHCO_3_, 5.56 mM glucose, and 10 mM HEPES, pH 7.4) was purchased from Life Technologies (Grand Island, NY, USA). HBSS without Ca^2+^ and Mg^2+^ is designated as HBSS^–^; HBSS containing 1.3 mM CaCl_2_ and 1.0 mM MgSO_4_ is designated as HBSS^+^.

### 3.3. Essential Oil Extraction

Essential oils were obtained by hydrodistillation of dried plant material using a Clevenger-type apparatus, as previously described [86]. We used conditions accepted by the European Pharmacopoeia (European Directorate for the Quality of Medicines, Council of Europe, Strasbourg, France, 2014) to avoid artifacts. The essential oils collected in the graduated tube of the Clevenger-type apparatus were stored in amber vials and used for subsequent analyses. Yields were calculated based on the amount of air-dried plant material used. Stock solutions of the essential oils were prepared in DMSO (10 mg/mL) for biological evaluation and in *n*-hexane (10% *w/v*) for gas-chromatographic analysis.

### 3.4. Gas Chromatography-Mass Spectrometry (GC-MS) Analysis

GC-MS analysis was performed with an Agilent 5975 GC-MSD system (Agilent Technologies, Santa Clara, CA, USA), as reported previously [99]. An Agilent Innowax FSC column (60 m × 0.25 mm, 0.25 μm film thickness) was used with He as the carrier gas (0.8 mL/min). The GC oven temperature was kept at 60 °C for 10 min, increased to 220 °C at a rate of 4 °C/min, kept constant at 220 °C for 10 min, and then increased to 240 °C at a rate of 1 °C/min. The split ratio was adjusted to 40:1, and the injector temperature was 250 °C. MS spectra were monitored at 70 eV with a mass range of 35 to 450 *m*/*z*. GC analysis was carried out using an Agilent 6890N GC system. To obtain the same elution order as with GC-MS, the line was split for FID and MS detectors, and a single injection was performed using the same column and appropriate operational conditions. Flame ionization detector (FID) temperature was 300 °C. The essential oil components were identified by co-injection with standards (whenever possible), which were purchased from commercial sources or isolated from natural sources. In addition, compound identities were confirmed by comparison of their mass spectra with those in the Wiley GC/MS Library (Wiley, NY, USA), MassFinder software 4.0 (Dr. Hochmuth Scientific Consulting, Hamburg, Germany), Adams Library, and NIST Library. Confirmation was also achieved using the in-house “Başer Library of Essential Oil Constituents” database, obtained from chromatographic runs of pure compounds performed with the same equipment and conditions. A C_8_–C_40_ *n*-alkane standard solution (Fluka, Buchs, Switzerland) was used to spike the samples for the determination of relative retention indices (RRI). Relative percentage amounts of the separated compounds were calculated from the FID chromatograms.

### 3.5. Isolation of Human Neutrophils

For isolation of human neutrophils, blood was collected from healthy donors in accordance with a protocol approved by the Institutional Review Board at Montana State University (Protocol #MQ041017). Neutrophils were purified from the blood using dextran sedimentation, followed by Histopaque 1077 gradient separation and hypotonic lysis of red blood cells, as described previously [100]. Isolated neutrophils were washed twice and resuspended in HBSS^–^. Neutrophil preparations were routinely >95% pure, as determined by light microscopy, and >98% viable, as determined by trypan blue exclusion. Neutrophils were obtained from multiple different donors (*n* = 8); however, the cells from different donors were never pooled during experiments.

### 3.6. Cell Culture

Human promyelocytic leukemia HL60 cells stably transfected with FPR1 (FPR1-HL60 cells) or FPR2 (FPR2-HL60 cells) (kind gifts from Dr. Marie-Josephe Rabiet, INSERM, Grenoble, France) were cultured in RPMI-1640 medium supplemented with 10% heat-inactivated fetal calf serum, 10 mM HEPES, 100 μg/mL streptomycin, 100 U/mL penicillin, and G418 (1 mg/mL). G418 was removed in the last round of culture before assays were performed.

### 3.7. Ca^2+^ Mobilization Assay

Changes in intracellular Ca^2+^ concentrations ([Ca^2+^]_i_) were measured with a FlexStation 3 scanning fluorometer (Molecular Devices, Sunnyvale, CA, USA). Briefly, human neutrophils were suspended in HBSS^-^, loaded with Fluo-4AM at a final concentration of 1.25 μg/mL, and incubated for 30 min in the dark at 37 °C. After dye loading, the cells were washed with HBSS^-^, resuspended in HBSS^+^, separated into aliquots, and loaded into the wells of flat-bottom, half-area well black microtiter plates (2 × 10^5^ cells/well). To assess the direct effects of test compound or pure essential oils on Ca^2+^ flux, the compound/oil was added to the wells (final concentration of DMSO was 1%), and changes in fluorescence were monitored (λ_ex_ = 485 nm, λ_em_ = 538 nm) every 5 s for 240 s at room temperature after addition of the test compound. To evaluate inhibitory effects of the compounds on FPR1/FPR2-dependent Ca^2+^ flux, the compound/oil was added to the wells (final concentration of DMSO was 1%) with cells (human neutrophils or FPR1/FPR2 HL60 cells). The samples were preincubated for 10 min, followed by addition of 5 nM *f*MLF (for human neutrophils or FPR1-HL60 cells) or 5 nM WKYMVM (for FPR2-HL60 cells). The maximum change in fluorescence, expressed in arbitrary units over baseline, was used to determine the agonist response. Responses were normalized to the response induced by 5 nM *f*MLF or 5 nM WKYMVM, which were assigned as 100%. Curve fitting (at least five or six points) and calculation of median effective concentration values (EC_50_ or IC_50_) were performed by nonlinear regression analysis of the dose–response curves generated using Prism 9 (GraphPad Software, Inc., San Diego, CA, USA).

### 3.8. Chemotaxis Assay

Human neutrophils were resuspended in HBSS^+^ containing 2% (*v/v*) heat-inactivated fetal bovine serum (2 × 10^6^ cells/mL), and chemotaxis was analyzed in 96-well ChemoTx chemotaxis chambers (Neuroprobe, Gaithersburg, MD, USA). In brief, neutrophils were preincubated with the indicated concentrations of the test sample (essential oil or pure compound) or DMSO (1% final concentration) for 30 min at room temperature and added to the upper wells of the ChemoTx chemotaxis chambers. The lower wells were loaded with 30 µL of HBSS^+^ containing 2% (*v/v*) fetal bovine serum and the indicated concentrations of test sample, DMSO (negative control), or 1 nM *f*MLF as a positive control. Neutrophils were added to the upper wells and allowed to migrate through the 5.0-µm pore polycarbonate membrane filter for 60 min at 37 °C and 5% CO_2_. The number of migrated cells was determined by measuring ATP in lysates of transmigrated cells using a luminescence-based assay (CellTiter-Glo; Promega, Madison, WI, USA), and luminescence measurements were converted to absolute cell numbers by comparison of the values with standard curves obtained with known numbers of neutrophils. Curve fitting (at least eight to nine points) and calculation of median effective concentration values (IC_50_) were performed by nonlinear regression analysis of the dose-response curves generated using GraphPad Prism 9.

### 3.9. Cytotoxicity Assay

Cytotoxicity of essential oils and pure compounds in HL60 monocytic cells was analyzed with a CellTiter-Glo Luminescent Cell Viability Assay Kit (Promega), according to the manufacturer’s protocol. Briefly, HL60 cells were cultured at a density of 10^4^ cells/well with different concentrations of essential oil or compound (final concentration of DMSO was 1%) for 30 min or 2 h at 37 °C and 5% CO_2_. Following treatment, substrate was added to the cells, and the samples were analyzed with a Fluoroscan Ascent FL microplate reader.

### 3.10. Molecular Modeling

The PharmMapper Server [101] was used for identifying the protein targets for (+)-cedrol. For a given small molecule, PharmMapper recognizes potential target possibilities using an “invert” pharmacophore mapping methodology. In several reference databases which are incorporated in the software, the protein biotargets are represented by sets of pharmacophore points that provide faster mapping. The PubChem database (https://pubchem.ncbi.nlm.nih.gov; accessed on 20 September 2021) was used as a source of initial 3D structures of the investigated compounds. The structure of (+)-cedrol (CID: 65575) was downloaded from PubChem in SDF format and further uploaded into the PharmMapper web server. Up to 300 conformers of each compound were automatically generated using a corresponding option of the software. The pharmacophore mapping was performed with the “Human Protein Targets Only” database containing 2241 targets. The top 250 potential targets per compound were retrieved and sorted by the normalized fit score. The physicochemical properties of (+)-cedrol were computed using SwissADME (http://www.swissadme.ch; accessed on 20 September 2021).

### 3.11. Statistical Analysis

One-way analysis of variance (ANOVA) was performed on the data sets, followed by Tukey’s pair-wise comparisons. Pair-wise comparisons with differences at *p* < 0.05 were considered to be statistically significant.

## 4. Conclusions

Compositional analysis of 16 essential oils isolated from Juniper plants revealed similarities and differences between our analyses and those previous reported for essential oils from this species. Notably, our studies represent the first time that essential oils isolated from the cones of many of these Juniper species have been analyzed, including cones from *J. sabina* (JSA_Con_), *J. pseudosabina* (JPS_Con_), and *J. pseudosabina* subsp. *turkestanica* (JT_Con_). Interestingly, several of the essential oil samples from Juniper collected in Kazakhstan but not in Montana contained high levels of cedrol, which was fairly unique among the samples and was enriched in JSE, JSA, and JPS. Evaluation of the biological activities of the parent essential oils or pure cedrol itself showed that they induced intracellular Ca^2+^ mobilization in human neutrophils, which is a key component of neutrophil activation. Indeed, pretreatment of cells with these essential oils or cedrol inhibited subsequent heterologous agonist-induced Ca^2+^ mobilization, which is a common feature of many neutrophil agonists. Likewise, pretreatment with JSE_Con_ (16.8% cedrol content) or pure cedrol inhibited human neutrophil chemotaxis toward *N*-formyl peptide. Thus, these data suggest that cedrol is a neutrophil agonist that can desensitize cells to subsequent stimulation by N-formyl peptide. However, further studies are necessary to define the precise mechanisms involved in the anti-inflammatory properties of cedrol.

## Figures and Tables

**Table 1:** Location and date of collection of the plant material.

*Juniperus* spp.	Locality	Latitude (N)	Longitude (E)	Altitude (m)	Plant Material	Date of Collection	Yield (%) Leaves/Cones
*Juniperus pseudosabina* Fisch.	Trans-Ili Alatau, Big Almaty gorge, Almaty region, South-Eastern Kazakhstan	43.04450°	76.97850°	2714	leaves	July 2019	0.5
*Juniperus pseudosabina* Fisch.	Trans-Ili Alatau, Kim-Asar gorge, Almaty region, South-Eastern Kazakhstan	43.16250°	77.09388°	2264	cones	September 2020	0.7
*Juniperus sibirica* Burgsd.	Trans-Ili Alatau, Big Almaty gorge, Almaty region, South-Eastern Kazakhstan	43.04450°	76.97850°	2714	leaves	September 2020	0.7
*Juniperus sibirica* Burgsd.	Lineisky Ridge, West Altai Nature Reserve, Eastern Kazakhstan	49.2544°	82.5736°	1589	cones	August 2020	0.8
*Juniperus pseudosabina* subsp. *turkestanica* Kom.	Western Tien-Shan, Aksu-Zhabagly Nature Reserve, sublatitudinal crest of the ridge in the region of the Kshi-Kaindy pass, Turkistan region, Southern Kazakhstan	42.39352°	70.55950°	1854	leaves/cones	July 2020	0.4/0.6
*Juniperus seravschanica* Kom.	Western Tien-Shan, Mashat gorge, Aksu-Zhabagly Nature Reserve, Turkistan region, Southern Kazakhstan	42.41652°	70.20741°	1005	leaves/cones	August 2021	0.5/0.4
*Juniperus sabina* L.	Trans-Ili Alatau, Kim-Asar gorge, Almaty region, South-Eastern Kazakhstan	43.16250°	77.09388°	2264	leaves/cones	September 2020	1.4/2.0
*J. horizontalis*	Bozeman, MT, USA	45.66885°	111.06629°	1462	leaves/cones	August 2019	1.0/1.2
*J. scopolorum*	Sypes canyon, Bozeman, MT, USA	45.74118°	110.98698°	1415	leaves/cones	August 2019	1.1/1.0
*J. communis*	Hyalite Canyon, Bozeman, MT, USA	45.48873°	111.00474°	2142	leaves/cones	August 2019	0.6/1.0

**Table 2:** Main compound components of essential oils (%) isolated from leaves and cones of different *Juniperus* species.

No	RRI	Compound	JH_Lv_	JH_Con_	JS_Lv_	JS_Con_	JC_Lv_	JC_Con_	JSE_Lv_	JSE_Con_	JSA_Lv_	JSA_Con_	JT_Lv_	JT_Con_	JPS_Lv_	JPS_Con_	JSI_Lv_	JSI_Con_
1	1032	α-Pinene	1.2	**3.0**	1.3	**4.0**	**68.7**	**22.3**	**45.3**	**34.4**	1.2	**4.3**	**15.2**	**25.1**	**30.8**	**49.3**	**26.9**	**44.9**
2	1035	α-Thujene	0.5	1.8	1.2	**2.3**					0.7	1.8	1.1	1.8			1.7	
3	1118	β-Pinene	0.1	0.2	0.1	0.2	**3.3**	0.6	0.7	0.6	0.1	0.2	1.5	1.6	**2.8**	**3.7**	1.8	1.1
4	1132	Sabinene	**17.3**	**77.4**	**37.6**	**35.8**	0.4	0.2	0.2	0.4	**33.2**	**74.1**	**39.6**	**54.2**	**19.4**	**20.4**	**24.3**	0.3
5	1159	δ-3-Carene					**2.2**	t					0.8	t	0.1		1.3	0.4
6	1174	Myrcene	**9.8**	**5.3**	0.9	**3.5**	**3.3**	**53.2**	**19.7**	**19.5**	**2.7**	**5.5**	**3.2**	**4.8**	**2.0**	**4.1**	**2.6**	**2.8**
7	1188	α-Terpinene	0.6	0.5	1.2	**2.5**	t		0.1	0.1	0.3	0.3	1.5	0.3	0.7	0.6	1.3	
8	1203	Limonene	**4.6**	1.5					**3.2**	**4.5**	1.2	1.2	1.0	1.2	0.8	1.0	1.8	
9	1255	γ-Terpinene	1.0	0.9	**2.1**	**4.8**	t	t	0.7	1	0.6	0.6	**2.6**	0.5	1.1	1.0	**2.2**	t
10	1280	*p*-Cymene	t	0.1	1.1	**2.5**	t	t	0.9	0.2	0.4	0.1	0.2	0.6	0.4	0.2	1.1	t
11	1451	β-Thujone	**2.6**	t					0.1		0.9	t	0.1		0.1		0.4	
12	1553	Linalool	0.2	0.6	0.1	0.1	0.1	0.6	1.3		1.0	1.8	**3.6**	0.1	**2.5**	0.3	1.0	
13	1579	Pregeijerene B	0.1		**2.2**	0.2												
14	1590	Bornyl acetate	**26.3**	t	0.1	t	0.6	0.3	0.7	1.2	0.1	t	0.1	0.1	t	0.4	0.2	0.4
15	1611	Terpinen-4-ol	**3.9**	**3.1**	**10.0**	**23.0**	0.1	0.2	0.5	1.3	**3.2**	1.5	**9.5**	**2.7**	**4.0**	**2.7**	**9.3**	0.8
16	1658	Sabinyl acetate	**16.8**		t						**30.3**	1.0		t		t		
17	1709	α-Terpinyl acetate		0.2				0.2			0.3	0.4					**2.7**	0.7
18	1726	Germacrene D	0.2	0.6	0.1	0.6	**3.9**	**8.7**	0.1	0.9		0.1		0.2		0.4	0.4	**16.7**
19	1773	δ-Cadinene	0.9	0.1	0.8	0.3	0.4	0.7	0.3	0.2	0.1	t	0.7	0.1	0.6	1	0.8	**5.8**
20	2069	1,6-Germacradien-5β-ol			0.7		0.5	1.1	0.1	0.2	0.2	0.1	0.7	0.3	0.7	1.4	0.8	**4.2**
21	2096	Elemol	**2.1**	0.1	**14.4**	**6.0**				0.5			**2.9**	1.3	**3.9**	1.2	0.7	0.3
22	2143	Cedrol							**13.1**	**16.8**	**12.2**	1.0		t	**7.0**	**3.4**		
23	2479	8-α-Acetoxyelemol	0.1		**11.1**	1.0							t	t				
24	2492	8,13-Abietadiene	0.1	0.1		0.4			0.1	**3.9**	0.2		0.4	0.1	0.4		t	

The data are presented as relative % for each component that was identified in the essential oils. RRI, relative retention index calculated on the basis of retention of n-alkanes; %, calculated from flame ionization detector data. Trace amounts (t) were present at <0.1%. All other compounds were identified by comparison with co-injected standards. Major component compounds (>2%) are indicated in bold. Abbreviations: JH_Lv_, *J. horizontalis* leaves; JH_Con_, *J. horizontalis* cones; JS_Lv_, *J. scopolorum* leaves; JS_Con_, *J. scopolorum* cones; JC_Lv_, *J. communis* leaves; JC_Con_, *J. communis* cones; JSE_Lv_, *J. seravschanica* leaves; JSE_Con_, *J. seravschanica* cones; JSA_Lv_, *J. sabina* leaves; JSA_Con_, *J. sabina* cones; JT_Lv_; *J. pseudosabina* subsp. *turkestanica* leaves; JT_Con_, *J. pseudosabina* subsp. *turkestanica* cones; JPS_Lv_, *J. pseudosabina* leaves; JPS_Con_, *J. pseudosabina* cones; JSI_Lv_, *J. sibirica* leaves; JSI_Con_, *J. sibirica* cones.

**Table 3:** Summary of the chemical composition (%) of essential oils from *Juniperus* species.

Compounds	JH_Lv_	JH_Con_	JS_Lv_	JS_Con_	JC_Lv_	JC_Con_	JSE_Lv_	JSE_Con_	JSA_Lv_	JSA_Con_	JT_Lv_	JT_Con_	JPS_Lv_	JPS_Con_	JSI_Lv_	JSI_Con_
Monoterpene hydrocarbons	37.8	91.9	85.0	59.6	84.9	77.9	72.0	62.2	41.0	89.4	68	91.1	59.3	82.3	68.6	50.3
Oxygenated monoterpenes	51.8	6.4	13.3	28.5	4.8	4.9	4.4	3.2	42.6	7.0	20.1	4.4	20.2	4.6	23.0	2.2
Sesquiterpene hydrocarbons	1.5	0.9	1.4	1.2	7.3	14.6	6.9	7.0	1.5	0.6	1.2	0.9	1.9	3.7	3.2	33.5
Oxygenated sesquiterpenes	5.1	0.1	32.6	9.0	1.7	1.9	13.7	18.1	12.5	1.2	6.4	2.7	14.0	8.5	3.9	12.9
All sesquiterpenes	6.6	1.0	34.0	10.2	9.0	16.5	20.6	25.0	14.0	1.8	7.6	3.6	15.9	12.2	7.1	46.4
Diterpenes	0.5	0.1		0.4		0.4	0.1	5.4	0.4		0.4	0.1	0.6			0.2
Fatty acids					0.2		0.2				0.1	0.1	0.8	0.1	0.2	
Others		0.1	2.2	0.2	0.4		0.1		0.3	0.1	2.7	0.3	1.7	0.2	0.1	
Total	96.7	99.5	96.9	98.9	98.9	99.5	97.4	95.9	98.3	98.3	98.9	99.6	98.5	99.4	99.0	99.1

Abbreviations: JH_Lv_, *J. horizontalis* leaves; JH_Con_, *J. horizontalis* cones; JS_Lv_, *J. scopolorum* leaves; JS_Con_, *J. scopolorum* cones; JC_Lv_, *J. communis* leaves; JC_Con_, *J. communis* cones; JSE_Lv_, *J. seravschanica* leaves; JSE_Con_, *J. seravschanica* cones; JSA_Lv_, *J. sabina* leaves; JSA_Con_, *J. sabina* cones; JT_Lv_; *J. pseudosabina* subsp. *turkestanica* leaves; JT_Con_, *J. pseudosabina* subsp. *turkestanica* cones; JPS_Lv_, *J. pseudosabina* leaves; JPS_Con_, *J. pseudosabina* cones; JSI_Lv_, *J. sibirica* leaves; JSI_Con_, *J. sibirica* cones.

**Table 4:** Biological activity of essential oils isolated from leaves and cones of different *Juniperus* species, as well as pure cedrol.

Source of *Juniperus* Essential Oils	EO	Neutrophils	HL-60-FPR1	HL-60-FPR2	Neutrophils
		EC_50_ (μM)	IC_50_ (μM)		
*J. horizontalis* leaves	JH_Lv_	24.7 ± 6.2	11.8 ± 3.7	10.1 ± 2.4	13.8 ± 1.3
*J. horizontalis* cones	JH_Con_	49.8 ± 12.1	13.8 ± 4.4	18.7 ± 6.4	N.A.
*J. scopolorum* leaves	JSC_Lv_	24.8 ± 8.4	12.7 ± 3.6	12.8 ± 3.8	16.0 ± 2.9
*J. scopolorum* cones	JS_Con_	38.0 ± 9.4	12.1 ± 5.1	12.4 ± 4.5	27.9 ± 1.4
*J. communis* leaves	JC_Lv_	54.0 ± 3.5	10.1 ± 3.4	11.0 ± 4.5	34.7 ± 6.4
*J. communis* cones	JC_Con_	53.6 ± 1.9	13.6 ± 5.2	14.8 ± 4.3	29.2 ± 8.0
*J. seravschanica* leaves	JSE_Lv_	43.0 ± 7.7	11.6 ± 4.1	13.4 ± 3.5	35.5 ± 3.8
*J. seravschanica* cones	JSE_Con_	41.0 ± 7.1	16.0 ± 5.2	20.1 ± 6.8	34.2 ± 7.9
*J. sabina* leaves	JSA_Lv_	28.5 ± 9.3	13.6 ± 4.4	13.5 ± 4.2	23.6 ± 3.3
*J. sabina* cones	JSA_Con_	40.7 ± 4.3	11.8 ± 4.3	14.1 ± 2.5	35.0 ± 7.6
*J. pseudosabina* subsp. *turkestanica* leaves	JT_Lv_	43.0 ± 10.1	15.6 ± 5.5	15.4 ± 2.2	36.7 ± 10.8
*J. pseudosabina* subsp. *turkestanica* cones	JT_Con_	13.9 ± 4.2	14.1 ± 5.1	14.2 ± 2.8	29.7 ± 5.3
*J. pseudosabina* leaves	JPS_Lv_	43.5 ± 10.6	11.3 ± 4.4	12.0 ± 4.1	29.4 ± 10.2
*J. pseudosabina* cones	JPS_Con_	45.1 ± 9.7	14.9 ± 5.4	15.1 ± 4.2	36.5 ± 11.1
*J. sibirica* leaves	JSI_Lv_	34.3 ± 7.3	7.0 ± 2.3	16.5 ± 6.7	48.7 ± 8.2
*J. sibirica* cones	JSI_Con_	31.8 ± 3.8	10.2 ± 3.5	16.4 ± 4.9	20.3 ± 6.8
Cedrol		15.6 ± 2.5	54.0 ± 3.5	14.3 ± 3.5	15.4 ± 4.3

**Table 5:** Potential protein targets of (+)-cedrol identified by PharmMapper.

Rank	PDB ID	Target Name	Fit Score	Rank	PDB ID	Target Name	Fit Score
**1**	1REU	BMP2	1	11	2PIR	Androgen receptor	0.8213
**2**	1P49	Steroid sulfatase	1	12	3BL1	CA2	0.8032
**3**	1J96	AKR1C2	1	13	3CJG	VEGFR2	0.7553
**4**	1E7E	Serum albumin	1	14	2OF0	β-Secretase 1	0.75
**5**	1L6L	Apo A-II	1	15	1SQN	Progesterone receptor	0.75
**6**	1W8L	PPIase A	0.9633	16	2G01	JNK1	0.7472
**7**	2PG2	KIF11	0.9482	17	1ZXC	ADAM 17	0.7442
**8**	2C3I	Pim-1	0.8963	18	1SHL	Caspase-7	0.7399
**9**	1J78	DBP	0.8598	19	1P0P	Cholinesterase	0.7331
**10**	3EQM	P450 19A1	0.8397	20	1S95	PPP5	0.7326

Abbreviations: ADAM 17, metalloprotease 17; AKR1C2, aldo-keto reductase family 1 member C2 (bile acid binding protein); Apo A-II, apolipoprotein A-II; BMP2, bone morphogenetic protein 2; CA2, carbonic anhydrase 2; DBP, vitamin D-binding protein; KIF11, kinesin-like protein; JNK1, c-Jun N-terminal kinase 1; P450 19A1, cytochrome P450 19A1; Pim-1, proto-oncogene serine/threonine-protein kinase; PPIase A, peptidyl-prolyl cis-trans isomerase A; PPP5, serine/threonine-protein phosphatase 5; VEGFR2, vascular endothelial growth factor receptor 2.

**Table 6:** Predicted physicochemical properties of (+)-cedrol according to SwissADME results.

Molecular Descriptor	Property
Formula	C_15_H_26_O
M.W.	222.37
Heavy atoms	16
Fraction Csp^3^	1.00
Rotatable bonds	0
H-bond acceptors	1
H-bond donors	1
MR	68.56
tPSA	20.23
iLogP	2.99
BBB permeation	Yes

Abbreviations: M.W., molecular weight (g/mol); MR, molar refractivity; tPSA, topological polar surface area (Å^2^); iLogP, lipophilicity; BBB, blood–brain barrier.

## Data Availability

The data that support the findings of this study are available from the authors upon reasonable request.

## Supplementary Materials

The following are available online at Supplementary Table S1: Chemical composition of essential oils (%) isolated from leaves and cones of different Juniperus species.

## Sample Availability

Samples of the essential oils are available from the authors.

## Abrreviations

JHLv, *J. horizontalis* leaves; JHCon, *J. horizontalis* cones; JSLv, *J. scopolorum* leaves; JSCon, *J. scopolorum* cones; JCLv, *J. communis* leaves; JCCon, *J. communis* cones; JSELv, *J. seravschanica* leaves; JSECon, *J. seravschanica* cones; JSALv, *J. sabina* leaves; JSACon, *J. sabina* cones; JTLv; *J. turkestanica* leaves; JTCon, *J. turkestanica* cones; JPSLv, *J. pseudosabina* leaves; JPSCon, *J. pseudosabina* cones; JSILv, *J. sibirica* leaves; JSICon, *J. sibirica* cones.
