# Supplementary material for: Innate Immunomodulatory Activity of Cedrol, a Component of Essential Oils Isolated from Juniperus Species
Source: Molecules. 2021 Dec 16;26(24):7644. doi: 10.3390/molecules26247644 (PMC8709035; doi:10.3390/molecules26247644)
Supplement: Supplementary file 1 [file molecules-26-07644-s001.zip › molecules-1497133-supplementary.pdf]

Supplementary Materials

Innate Immunomodulatory Activity of Cedrol, a Component of Essential Oils Isolated From *Juniperus* Species

Gulmira Özek[1], Igor A. Schepetkin[2], Moldir Yermagambetova[3,4], Temel Özek[1,5], Liliya N. Kirpotina[2], Shyryn S. Almerekova[3,4], Saule I. Abugalieva[3,4], Andrei I. Khlebnikov[6], and Mark T. Quinn[2]

[1]*Department of Pharmacognosy, Faculty of Pharmacy, Anadolu University, Eskisehir, 26470, Turkey*

[2]*Department of Microbiology and Cell Biology, Montana State University, Bozeman, MT 59717, United States*

[3]*Institute of Plant Biology and Biotechnology, Almaty 050040, Kazakhstan*

[4]*Faculty of Biology and Biotechnology, al-Farabi Kazakh National University, Almaty 050038, Kazakhstan*

[5]*Medicinal Plant, Drug and Scientific Research and Application Center (AUBIBAM), Anadolu University, 26470 Eskişehir, Turkey*

[6]*Kizhner Research Center, Tomsk Polytechnic University, Tomsk 634050, Russia*

Supplemental Table S1. Chemical composition of essential oils (%) isolated from leaves and cones of different Juniperus species. **Abbreviations**: JH_{Lv}, *J. horizontalis* leaves; JH_{Con}, *J. horizontalis* cones; JS_{Lv}, *J. scopolorum* leaves; JS_{Con}, *J. scopolorum* cones; JC_{Lv}, *J. communis* leaves; JC_{Con}, *J. communis* cones; JSE_{Lv}, *J. seravschanica* leaves; JSE_{Con}, *J. seravschanica* cones; JSA_{Lv}, *J. sabina* leaves; JSA_{Con},

J. sabina cones; JT$_{Lv}$; *J. turkestanica* leaves; JT$_{Con}$, *J. turkestanica* cones; JPS$_{Lv}$, *J. pseudosabina* leaves; JPS$_{Con}$, *J. pseudosabina* cones; JSI$_{Lv}$, *J. sibirica* leaves; JSI$_{Con}$, *J. sibirica* cones.

#	RRI	Compound	JH$_{Lv}$	JH$_{Con}$	JS$_{Lv}$	JS$_{Con}$	JC$_{Lv}$	JC$_{Con}$	JSE$_{Lv}$	JSE$_{Con}$	JSA$_{Lv}$	JSA$_{Con}$	JT$_{Lv}$	JT$_{Con}$	JPS$_{Lv}$	JPS$_{Con}$	JSI$_{Lv}$	JSI$_{Con}$
1	1014	Tricyclene	0.9					t	0.1	0.1		t	t	t	t	t	0.1	t
2	1032	**α-Pinene**	1.2	**3.0**	1.3	**4.0**	**68.7**	**22.3**	**45.3**	**34.4**	1.2	**4.3**	**15.2**	**25.1**	**30.8**	**49.3**	**26.9**	**44.9**
3	1035	α-Thujene	0.5	1.8	1.2	**2.3**					0.7	1.8	1.1	1.8			1.7	
4	1072	α-Fenchene				t	0.1		0.1	t		t	t	t	t	t	0.1	t
5	1076	Camphene	0.9			0.1	0.4	t	0.3	0.1		0.1	0.1	0.2	0.3	0.3	0.3	0.2
6	1088	Isopropyl isovalerate													t	t	t	
7	1093	Hexanal													t		0.1	t
8	1118	β-Pinene	0.1	0.2	0.1	0.2	3.3	0.6	0.7	0.6	0.1	0.2	1.5	1.6	**2.8**	**3.7**	1.8	1.1
9	1132	**Sabinene**	**17.3**	**77.4**	**37.6**	**35.8**	0.4	0.2	0.2	0.4	**33.2**	**74.1**	**39.6**	**54.2**	**19.4**	**20.4**	**24.3**	0.3
10	1133	**δ-4-Carene**					0.1											
11	1151	***p*-Xylene**					t											
12	1157	***m*-Xylene**					t											
13	1158	Thuja-2,4(10)-diene											t		0.2	t	0.1	
14	1159	δ-3-Carene					**2.2**	t					0.8	t	0.1		1.3	0.4
15	1174	**Myrcene**	**9.8**	**5.3**	0.9	**3.5**	**3.3**	**53.2**	**19.7**	**19.5**	**2.7**	**5.5**	**3.2**	**4.8**	**2.0**	**4.1**	**2.6**	**2.8**
16	1176	α-Phellandrene	t	t	t	0.1	0.1	t		t	t	t	t	t	t	t	0.3	0.2
17	1188	α-Terpinene	0.6	0.5	1.2	**2.5**	t		0.1	0.1	0.3	0.3	1.5	0.3	0.7	0.6	1.3	
18	1202	Sylvestrene					t											
19	1203	Limonene	**4.6**	1.5					**3.2**	**4.5**	1.2	1.2	1	1.2	0.8	1	1.8	
20	1213	1,8-Cineole											1.1					
21	1218	β-Phellandrene		t	0.1	0.3	0.8		0.2	0.1	t	t	t	0.3	0.4	0.9	1.4	
22	1240	p-Mentha-1,3,6-triene													t		t	
23	1225	(*Z*)-3-Hexenal					0.1	t	t								t	
24	1244	Amyl furan					t											
25	1246	(*Z*)- β-Ocimene					t	t										
26	1255	γ-Terpinene	1	0.9	**2.1**	**4.8**	t	t	0.7	1.0	0.6	0.6	**2.6**	0.5	1.1	1.0	**2.2**	t
27	1266	(*E*)- β-Ocimene	0.1	0.1			t	t	t		0.2	0.1						
28	1266	3-Octanone					t											
29	1280	*p*-Cymene	t	0.1	1.1	**2.5**	t	t	0.9	0.2	0.4	0.1	0.2	0.6	0.4	0.2	1.1	t
30	1286	Isoterpinolene					t											
31	1290	Terpinolene	0.8	1.1	0.9	1.9	0.6	0.4	0.5	1.2	0.4	1.1	1.1	0.5	0.3	0.7	1.2	0.4
32	1299	2-Methylbutyl isovalerate					t						t			0.1	t	
33	1348	6-Methyl-5-hepten-2-one													t			
34	1360	Hexanol							0.1									
35	1362	*cis*-Rose oxide									0.1							

36	1379	3-Methyl-3-butenyl isovalerate					t			t			0.1	0.1	0.3		0.2	
37	1384	α-Pinene oxide							0.1	t				t				
38	1386	Octenyl acetate					t											
39	1393	3-Octanol					t											
40	1398	2-Nonanone									0.2		**2.2**	0.1	1.0			
41	1400	Nonanal					t				t							
42	1402	(*E*,*Z*)-1,3,5-Undecatriene		t			t											
43	1406	α-Fenchone							0.4	t								
44	1408	1,3,8-*p*-Menthatriene		t			t			t								
45	1418	*o*-Methylanisol					0.1											
46	1429	Perillene							0.1	t					0.1			
47	1437	α-Thujone	0.2								0.1						0.1	
48	1439	γ-Campholene aldehyde													0.1			t
49	1443	2,5- Dimethylstyrene								t	t						0.1	t
50	1446	1-Octen-3-ol					0.2											
51	1450	*trans*-Linalool oxide		t				0.1	t		0.1	0.1	t					
52	1451	β-Thujone	**2.6**	t					0.1		0.9	t	0.1		0.1		0.4	
53	1452	α,*p*-Dimethylstyrene		t					t				0.1			0.1		
54	1457	Hexyl-3-methyl butyrate							t									
55	1466	α-Cubebene		t			t	t									t	0.2
56	1468	*trans*-1,2-Limonene epoxide							0.1									
57	1474	*trans*-Sabinene hydrate		1.3	0.7	0.7	t	0.2		0.1	0.3	0.6	0.4	0.3	0.5	0.1	0.8	t
58	1477	4,8-Epoxyterpinolene									t						t	
59	1479	δ-Elemene		0.1						0.2		t		t		0.2		t
60	1482	*cis*-Linalool oxide						t				t	t		t			
61	1482	Fenchyl acetate	t	t			t			0.1						t		
62	1487	Citronellal					0.3	0.5			t		0.6				t	
63	1494	(*Z*)-3-Hexenyl 3-methylbutyrate													0.1			
64	1495	Bicycloelemene																t
65	1497	α-Copaene	t				t	t	t									0.1
66	1499	α-Campholene aldehyde					0.1								0.7		0.3	t
67	1521	2-Nonanol													0.4			
68	1525	α-Funebrene							t									
69	1532	Camphor	0.2						0.4									t
70	1535	β-Bourbonene					t											t
71	1536	Pinocamphone													0.1		0.1	
72	1549	β-Cubebene					t											0.1
73	1551	7-*epi*-Sesquithujene								0.1	t							

74	1553	Linalool	0.2	0.6	0.1	0.1	0.1	0.6	1.3		1	1.8	**3.6**	0.1	**2.5**	0.3	1	
75	1556	*cis*-Sabinene hydrate		0.7	0.7	0.7				t	0.2	0.4	0.4	0.3	0.6	0.1	0.6	
76	1562	Octanol					t											
77	1565	Linalyl acetate									0.4	0.7		t			t	
78	1565	Methyl citronellate	0.1	0.1	0.2	0.1	0.1	0.7			0.3	t	0.4	0.1	t	t	0.3	0.3
79	1571	*trans*-*p*-Menth-2-en-1-ol		0.2	0.6	1.2	t			0.1	0.2	0.1	0.5	0.2	0.3	0.2	0.6	t
80	1574	Acetoxy linalloloxide										t						
81	1577	α-Cedrene							0.3	0.2	0.1	0.1			t			
82	1579	Pregeijerene B	0.1		**2.2**	0.2												
83	1586	Pinocarvone													0.3		t	
84	1587	1,7-Diepi-β-cedrene							1.7	1.3	0.4				0.2	0.2		0.2
85	1589	β-Ylangene					0.1											
86	1590	**Bornyl acetate**	**26.3**	t	0.1	t	0.6	0.3	0.7	1.2	0.1	t	0.1	0.1	t	0.4	0.2	0.4
87	1594	*trans*-β-Bergamotene							0.1	0.1								
88	1600	β-Elemene		t		t	0.8	0.3		0.2		t		0.1		0.2	0.5	1.6
89	1604	Thymol methyl ether								t	t							
90	1604	2-Undecanone		0.1							0.1	0.1	0.4	0.2	0.2	0.1		
91	1604	Isobornyl acetate	0.5															
92	1608	β-Copaene					t											t
93	1608	Camphene hydrate	t												t			
94	1611	**Terpinen-4-ol**	**3.9**	**3.1**	**10.0**	**23.0**	0.1	0.2	0.5	1.3	**3.2**	1.5	**9.5**	**2.7**	**4.0**	**2.7**	**9.3**	0.8
95	1612	β-Caryophyllene					0.2	0.4		t								0.8
96	1613	β-Cedrene							0.3		0.1	t			t	t		
97	1614	2-Methyl-6-methylene-3,7-octadiene-2-ol*													0.1			
98	1614	Sylveterpenyl acetate					0.2											
99	1617	Hexyl hexanoate							0.1									
100	1628	Aromadendrene																t
101	1630	Terpinen-4-yl acetate		t												t	0.3	
102	1638	*cis*-*p*-Menth-2-en-1-ol		0.1		0.9	t				0.2	t	0.4	0.1	0.2	0.1	0.4	
103	1644	Widdrene (Thujopsene)							0.4	0.3	0.3				0.2	0.1		
104	1645	*cis*-Verbenyl acetate					0.2											
105	1648	Myrtenal							t						0.3		0.1	
106	1650	γ-Elemene		t				0.1	0.1	0.3				0.1		0.2	0.1	
107	1629	(Z)-Ocimenyl acetate	0.1															
108	1651	Sabinaketone															0.2	
109	1652	*trans*-Linalool oxide acetate			t													
110	1658	**Sabinyl acetate**	**16.8**		t						**30.3**	1		t		t		

111	1659	γ-Gurjunene					t											
112	1661	*trans*-Pinocarvyl acetate					t											
113	1663	*cis*-Verbenol													0.3			
114	1668	Citronellyl acetate					0.3	0.2										
115	1668	(*Z*)-β-Farnesene		t			t	0.3	0.4	0.3		0.1		t	0.3	0.1		0.4
116	1670	*trans*-Pinocarveol					t		0.1	t					1.6		0.5	
117	1677	*epi*-Zonarene			t		t									t		0.1
118	1680	α-Acoradiene							0.1	0.1								
119	1682	*trans*-Verbenol											t		**3.3**	0.2	0.6	
120	1683	Bicyclosesquiphellandrene								0.2								
121	1685	Cadina-3,5-diene	0.1													t		
122	1687	α-Humulene					0.4	0.6									0.6	1.9
123	1689	*trans*-Piperitol		t	0.2	0.3					0.1		0.2	0.1	0.1			
124	1690	Cryptone													0.1		0.2	
125	1692	(*E*)-Methyl geranate										0.2	0.3	0.1	0.1			
126	1693	β-Acoradiene							0.2	0.1								
127	1696	γ-Terpinyl acetate					0.4											
128	1700	Limonen-4-ol	t								0.1		0.1					
129	1704	(*Z*)-Ocimenol			0.4													
130	1704	Myrtenyl acetate															0.3	
131	1704	γ-Curcumene								t								
132	1704	γ-Muurolene	t						0.1	t						0.1		0.6
133	1706	α-Terpineol	0.2			0.9	0.2	0.6	0.2	0.1			0.4	0.1	0.3	0.3		
134	1705	(*E*)-Methyl geranate		0.1							1.7	0.2						
135	1709	α-Terpinyl acetate		0.2				0.2			0.3	0.4					**2.7**	0.7
136	1714	β-Alaskene							0.3	0.2		0.1						
137	1715	γ-Terpineol					0.2											
138	1718	Bicyclosesquiphellandrene										t	0.2					
139	1719	Borneol	0.3				t		0.2	0.1			0.3	t	1.2	0.2	0.5	t
140	1720	*trans*-Sabinol	0.2								1.8		0.1	t	0.2			
141	1720	β-Alaskene					t											
142	1724	β-Himachalene							t									
143	1726	α-Zingiberene								0.1								
144	1726	Germacrene D	0.2	0.6	0.1	0.6	**3.9**	**8.7**	0.1	0.9		0.1		0.2		0.4	0.4	**16.7**
145	1738	*p*-Mentha-1,5-dien-8-ol													1		0.6	
146	1738	β-Alaskene													0.1	t		
147	1740	α-Muurolene	0.1		0.2	0.2	0.1	0.1	0.1					0.1		0.2		1.4
148	1740	Valencene			0.2													

149	1741	β-Bisabolene					0.4	0.1	0.2	0.2	t	t						
150	1742	Geranial									0.1							
151	1742	β-Selinene							t							t	0.1	0.3
152	1743	α-Cadinene					t						t			0.1		0.3
153	1744	α-Selinene															0.1	0.2
154	1747	*trans*-Carvyl acetate					t											
155	1748	Piperitone	0.1						t		0.1							
156	1755	Bicyclogermacrene					0.4	0.2								0.1		0.9
157	1755	β-Curcumene							0.2			t	0.1					
158	1758	*cis*-Piperitol				0.5							0.3	0.1		t	0.2	
159	1759	α-Cuprenene									t							
160	1763	α-Alaskene							0.5	0.6	0.1	0.2				0.1		
161	1765	Geranyl acetate					0.2	0.5										
162	1771	g-Bisabolene					0.2		0.2	0.2		t						
163	1772	Citronellol	0.1	t	0.1		0.3	0.6			0.8		1.3		0.2	t		
164	1773	δ-Cadinene	0.9	0.1	0.8	0.3	0.4	0.7	0.3	0.2	0.1	t	0.7	0.1	0.6	1	0.8	**5.8**
165	1776	γ-Cadinene	0.1	t	0.1	0.1	0.2	0.2	0.2		t	t	0.1	t	0.2	0.2	0.2	1.4
166	1783	β-Sesquiphellandrene							0.2	0.3	0.1	t						
167	1784	(*E*)-α-Bisabolene					0.2											
168	1786	*ar*-Curcumene							0.3	t		t	0.1		0.3			
169	1796	Selina-3,7(11)-diene							0.2									
170	1799	Cadina-1,4-diene																0.1
171	1800	*cis*-Sabinol											t				0.2	
172	1801	β-Cuprenene								0.1	0.3	t						
173	1802	Hexyl octanoate							0.1									
174	1804	Myrtenol					0.2								0.7		1.3	
175	1805	α-Campholene alcohol					0.1		0.1								0.2	
176	1811	*p*-Mentha-1,3-dien-7-al									t		t		0.1		0.1	
177	1814	*p*-Mentha-1,5-dien-7-ol									t				0.3		0.1	
178	1815	2-Tridecanone											0.1			0.1		
179	1823	p-Mentha-1(7),5-dien-2-ol													0.2			
180	1827	(*E*,*E*)-2,4-Decadienal					t		0.1		t		t					
181	1845	*trans*-Carveol											t		0.4		0.2	
182	1849	Cuparene							0.2									
183	1853	*cis*-Calamenene																0.1
184	1854	Germacrene B		0.1				0.2	0.2	0.8		t	t	0.3		0.5	0.4	0.3
185	1856	*m*-Cymen-8-ol								0.1		t						
186	1857	Geraniol						0.2			0.1							
187	1864	*p*-Cymen-8-ol		t					t		0.1		t	0.1	0.3	t	0.6	

188	1870	Hexanoic acid					0.2											
189	1878	2,5-Dimethoxy-*p*-cymene					t											
190	1882	*cis-Carveol*													t			
191	1889	Myrtanol					t											
192	1895	Italicene ether													t			
193	1900	*epi*-Cubebol					t						t		0.1	t	t	0.3
194	1904	Elema-1,3-dien-6α-ol		t						0.1				t		0.1		
195	1957	Cubebol			0.1		t				t		t		t	t	t	0.3
196	1973	Dodecanol					t											
197	2003	(*E*)-2(3)-Dihydrofarnesal					0.1											
198	2008	Caryophyllene oxide															0.3	
199	2022	Fragranyl pentanoate			0.2													
200	2040	10-epi-Elemol			0.3													
201	2050	(*E*)-Nerolidol				0.4	0.2	0.5		0.1			t	t	0.1	0.1	0.2	
202	2069	1,6-Germacradien-5β-ol			0.7		0.5	1.1	0.1	0.2	0.2	0.1	0.7	0.3	0.7	1.4	0.8	**4.2**
203	2071	Humulene epoxide-II															0.2	t
204	2080	Cubenol	0.1										t			t		0.3
205	2088	1-*epi*-Cubenol	0.4										t					0.2
206	2092	β-Oplopenone			0.2										0.2			t
207	2095	Salviadienol																t
208	2096	Elemol	**2.1**	0.1	**14.4**	**6.0**				0.5			**2.9**	1.3	**3.9**	1.2	0.7	0.3
209	2104	Viridiflorol									t							
210	2108	Hedycaryol			0.1													
211	2134	Sesquithuriferol*										0.1						
212	2143	**Cedrol**							**13.1**	**16.8**	**12.2**	1		t	**7.0**	**3.4**		
213	2144	Spathulenol					**t**						t		0.1		0.1	0.1
214	2152	**α**-Acorenol								0.1					0.1	0.1		
215	2157	5-*epi*-*7*-*epi*-α-Eudesmol				0.1							t					
216	2178	Widdrol									t							
217	2184	*cis*-*p*-Menth-3-en-1,2-diol				0.1												
218	2185	γ-Eudesmol			1.3	0.8							0.5	0.2				
219	2187	Cedryl acetate							0.2									
220	2187	δ-Cadinol	0.4	t			0.1									0.4	0.3	1.3
221	2193	Eremoligenol											t					
222	2198	Thymol								0.1								
223	2202	**α**-Guaiol											t		t			
224	2209	δ-Muurolol	0.4	t	0.6	0.1		t	0.1		t		0.3	0.1	0.2	0.3	0.3	1.5
225	2219	δ-Cadinol	0.1		0.2		t		t		t		0.2			0.1		0.4
226	2219	Dimyrcene II-α						0.4		0.3								

227	2232	**α**-Bisabolol					0.5											
228	2250	**α**-Eudesmol	0.1	t	0.9	0.6				0.1			0.3	0.2	0.3	0.2	0.1	
229	2252	Citronellic acid					0.1											
230	2253	(6*E*)-2,3-Dihydrofarnesol											t					
231	2255	**α**-Cadinol	1.3	t	1.8		0.1	0.3	0.2	0.1	0.1		0.9	0.2	0.5	0.9	0.8	4
232	2257	β-Eudesmol	0.1	t	0.9					t			0.6	0.3	0.5	0.2	0.1	
233	2259	Guaia-3,9-dien-11-ol											t					
234	2260	Alismol		t						0.1	t		t	0.1		0.1		
235	2269	Dimyrcene II-β						t		0.1								
236	2273	Selin-11-en-4*α*-ol																t
237	2279	(2*Z*,6*E*)-Farnesal					t											
238	2341	(2*Z*,6*E*)-Farnesol					t											
239	2349	Isopimaradiene		t						0.1								
240	2367	Isopimara-8,15-diene								0.4		t						
241	2368	Eudesma-4(15),7-diene-1-β-ol													0.3			
242	2388	Dolabradiene		t														
243	2396	13-*epi*-Manoyl oxide		t						0.1								
244	2438	Kaur-16-ene								t								
245	2503	Dodecanoic acid													0.4		t	
246	2524	Abietatriene								0.2	t		t	t				0.2
247	2479	8-**α**-Acetoxyelemol	0.1		**11.1**	1.0							t	t				
248	2492	8,13-Abietadiene	0.1	0.1		0.4			0.1	**3.9**	0.2		0.4	0.1	0.4		t	
249	2498	Manool													0.2			
250	2582	Neoabietadiene*								0.3								
251	2830	4-*epi*-Abietal	0.2							t	t							
252	3049	Abieta-7,13-diene-3-one	0.2							t	0.2							

[a]The data are presented as relative % for each component that was identified in the essential oils. RRI, relative retention index calculated on the basis of retention of n-alkanes; %, calculated from flame ionization detector data. Trace amounts (t) were present at <0.1%. All other compounds were identified by comparison with co-injected standards. Major component compounds (>2%) are indicated in bold.
